# Exploratory visual analysis of conserved domains on multiple sequence alignments

**DOI:** 10.1186/1471-2105-10-S11-S7

**Published:** 2009-10-08

**Authors:** TJ Jankun-Kelly, Andrew D Lindeman, Susan M Bridges

**Affiliations:** 1 Institute for Digital Biology and Department of Computer Science and Engineering, Bagley College of Engineering, Mississippi State University, Mississippi, USA

## Abstract

**Background:**

Multiple alignment of protein sequences can provide insight into sequence conservation across many species and thus allow identification of those sections of the sequence most critical to protein function. This insight can be augmented by joint display of conserved domains along the sequences. By fusing this metadata visually, biologists can analyze sequence conservation and functional motifs simultaneously and efficiently.

**Results:**

We present MSAVis, a new approach combining luminance and hue for simultaneous visualization of conserved motifs and sequence alignment. Input for the algorithm is a multiple sequence alignment in a standard format. The NCBI Conserved Domain Database (CDD) is used for finding conserved domains along the alignment. The visualization quickly identifies conserved domains, and allows both macro (sequence-long) and micro (small amino-acid neighborhood) views.

**Conclusion:**

MSAVis utilizes two visual cues, luminance and hue, to facilitate at-a-glance summary of the conservation of a user-provided protein alignment while enabling multiple comparisons among functional domains. These visual cues are preattentive and separable so that the relationship between conservation strength and domain membership can be understood. The MSAVis software, written in Python and using BioPython and OpenGL, can be found at http://agbase.msstate.edu/tools/MSAVis.html.

## Background

**Figure 1:**
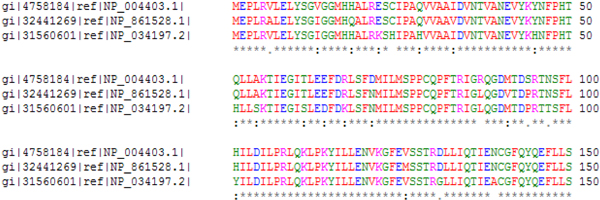
**Traditional depiction of multiple sequence alignment**. A depiction of the multiple sequence alignment of 3 DNA methyltransferase (Trdmt1) proteins from different organisms: Human [GenBank:NP_004403], cow [GenBank:NP_861528], and mouse [GenBank:NP_034197]. Strength of alignment is indicated by asterisks, colons, periods, or spaces from strongest to weakest. Clustal [1] was used to generate the image. In the image, color indicates physico-chemical properties of the amino-acid, and the symbols below each position in the sequence indicate the amount of conservation ('*': Exact, ':': Conserved Substitution, '.': Semi-conserved substitution).

*Multiple sequence alignment *(MSA) is one of the most widely used methods for assessing sequence conservation and conservaton of protein domains because it allows biologists to analyze the similarities and differences of related proteins at from the individual amino acid level to the sequence level. Figure 1 shows a portion of a multiple sequence alignment of DNA methyltransferase (Trdmt1) proteins from 3 different organisms: Human [GenBank:NP_004403], cow [GenBank:NP_861528], and mouse [GenBank:NP_034197]. The canonical representation of an MSA has each protein sequence on a separate line with matching characters aligned in columns and spaces inserted where necessary to improve the alignment – these spaces are called gaps and are represented by dashes. The amount of conservation of each position is represented by symbols at the bottom of each set of proteins: In the figure, an '*' (asterisk) represents an exact match in all sequences, a ':' (colon) means that a "conserved substitution has occurred" (i.e., the amino acids are not exactly the same, but are of the same general type), and a '.' (period) means that a "semi-conserved substitution has occurred" [1]. The degree of conservation can also be given as a number for either an individual sequence location or the entire sequence with larger numbers meaning a higher degree of conservation. Finally, as shown in the figure, color can be used to indicate the physico-chemical properties of the amino acid (e.g., blue for acidic); amino acids with similar properties share a hue. The similarities and differences highlighted by multiple sequence alignments can lead to conclusions about the evolutionary history of organisms, as well as information pinpointing functional parts of the sequences of each organism.

Sequence alignment provides information about the *primary structural similarity *of the proteins, however, this is not indicative by itself of their *functional similarity*. Biologists often want to investigate the functional domains of proteins. These are the sections of the proteins sequence that enable it to serve a particular biological role. Because these sections tend to be evolutionarily conserved (they remain the same in related organisms), they are also called *conserved domains *(CDs). NCBI states, "computational biologists define conserved domains based on recurring sequence patterns or motifs [2]." After a sequence has been identified as a functional conserved domain experimentally or using predictive methods, a computer model of the domain can be generated. That model is then used to identify the domain in sequences from other organisms. There are many online databases that take a protein sequence as a query and return matching domains; for this work, the Conserved Domain Database (CDD) from NCBI [2,3] was used.

**Figure 2:**
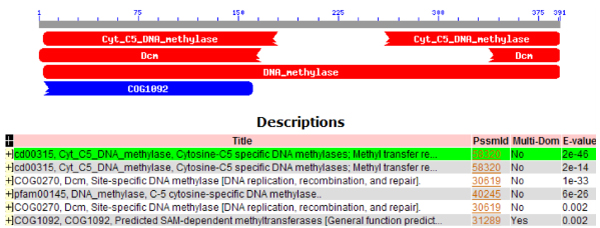
**Conserved domains over the human protein from Figure 1**. A depiction of all of the conserved domains from human Trdmt1 protein [GenBank:NP_004403]; each domain is represented by a blue/red ribbon over the sequence (gaps indicate gaps in the domain). Additional metadata about each domain is presented at the bottom. A figure like this can be generated for each protein to see all the conserved domains; comparison between them is difficult and inefficient. From NCBI's CDD [3].

As with MSAs, the conserved domains of a protein may be depicted as well (Figure 2). For a single protein, each domain is indicated by a bar corresponding to the regions where the domain is expressed; these can be continuous or segmented as shown in the figure. It is possible that the domain is incomplete if it is cut off at the beginning or the end of the protein sequence. This depiction provides a concise summary of the CDs for the given protein, but it is limited in its expressivity.

Both the MSA and CD diagrams provide clues to the similarities of proteins: The former indicates conservation of primary structure while the second shows inferred functional motifs. When these are viewed individually, however, it is difficult to determine how the primary structure and motifs interrelate. Sequence information shows the similarity of the multiple proteins, but no domain information. Similarly, domain information is typically only displayed for one protein at a time. To address these limitations, we have developed MSAVis, a method to visually fuse the strength of the alignment and the presence of conserved domains over a set of proteins. The remainder of this article reviews systems similar to MSAVis, and describes how MSAVis was designed using principles of visualization and its capabilities.

### Related work

Bioinformatics visualization presents inherently non-spatial biological data using interactive computer graphics. The purpose of these depictions is to solve a domain scientist's task. The variety of biovisualization systems is beyond the scope of our work; for a survey, see the summary by Lungu and Xu [4]. Instead, we review the work in sequence alignment and conserved domain visualization as a basis for our discussion about MSAVis.

As Figure 1 demonstrates, textual views of MSAs do not scale either in the number of proteins (rows per block) or in the length of the sequence (number of blocks). Graphical methods thus attempt to compress this information or provide methods for effective exploration. One of the first of these were Sequence Logos [5]. A sequence logo compresses the rows of the MSA diagram into a single display – the vertical column summarizes an entire group of proteins or nucleic acids (DNA or RNA). For each column (a position in the protein/nucleic acid string), a stack of letters that occur in that position is drawn; the letters are scaled by occurrence so that amino acids or nucleotides that occur more often are taller than those that occur infrequently. Sequence logos provide a quick glance at the most common characters amongst the sequences; however, the structure of an individual sequence is lost. Note that a sequence logo does not compress the length of the sequence, so scrolling is still required to view very long chains of characters.

In contrast to sequence logos, SequenceJuxtaposer [6] displays each character in every sequence over the entire length. However, it elides the display of the amino acid/nucleotide character by representing each by a unique color. Thus, it provides a zoomed out view of the entire sequence as a colored matrix where position (*i*, *j*) indicates the *j*th amino acid in protein *i*. The user can expand the display vertically (e.g, focusing on a subset of proteins) or horizontally (e.g., focusing on a region of the sequence). As the user zooms, the characters of the sequence are shown as space becomes available. Other methods compress the sequences graphically as well [7-9]. Some, like SequenceJuxtaposer, only show sequence information; others, like Phylo-Vista [8], also show related information (e.g., genomic data). None of them, however, fuse the conserved domain information with the sequences.

The area of conserved domain visualization is less explored. NCBI's Conserved Domain Database (CDD) provides a suite of tools for depicting CDs [2,3]. For a single protein, it produces images as demonstrated by Figure 2: All CDs over the protein are depicted. Similar, non-interactive views are available from InterPro [10,11] and UniProt [12,13]; proteins are shown (one at a time or in groups) with all the domains but no alignment details. This grouping prevents efficient visual comparison. In addition, CDD uses a phylogenetic tree-like visualization to show related domains from other species for a given selected CD. However, this tree view loses the sequence information from the original protein – one cannot view all the CDs from a group of proteins concurrently.

Jalview [14] is the tool that is most closely related to ours. Jalview can be used to both display and edit protein sequences, and can depict considerable metadata about a squence. Color is used in the sequence view to either show a single CD or the overall strength of the alignment; separate windows are used to breakdown the elements of the alignment strength. However, with Jalview it is not possible to effectively view more than a few domains on an alignment, especially if multiple conserved domains matches overlap on part of a sequence. Furthermore, it is not easily possible to get an overall view – across the entire alignment – of where each conserved domain lies as Jalview does not provide a compressed view as does SequenceJuxtaposer. Our MSAVis approach, though limited to MSA and CD visualization, visually fuses both in a manner not currently supported by extant tools; this visual fusion is explained in the next section.

### Implementation

MSAVis depicts aligned protein sequences, the strength of the alignment, and the conserved domains over the collected proteins. Starting from a pre-calculated alignment provided by the user in ClustalW [1] or PHYLIP [15] format, MSAVis queries the online NCBI CDD for each sequence and parses the results. Thus, for every position along a given sequence, the following is known:

• The protein to which the amino acid belongs

• Its position within the protein

• The strength of the overall alignment at the position over all the proteins

• Whether or not the position is part of one of the known conserved domains

**Figure 3:**
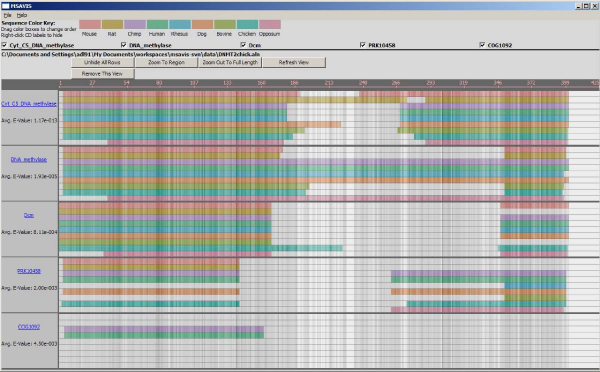
**The MSAVis visualization interface**. MSAVis visually fuses multiple sequence alignments with depictions of the conserved domains over the individual proteins. For each conserved domain, an alignment block is shown indicates where the conserved domain exists for the given protein (see Figure 4 for presentation details).

We use this information to build up the visualization (Figure 3). The elements of this visualization, and how one interacts with it, are discussed in this section.

### Visualization overview

**Figure 4:**
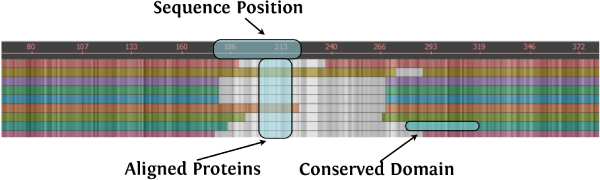
**Building block of the MSAVis visualization**. For each conserved domain, the sequence alignment information is repeated: Each protein is represented as a row where columns are shaded by alignment strength (stronger alignment is darker). Regions of a row that are colored by a color unique to the protein are part of the given conserved domain.

**Figure 5:**
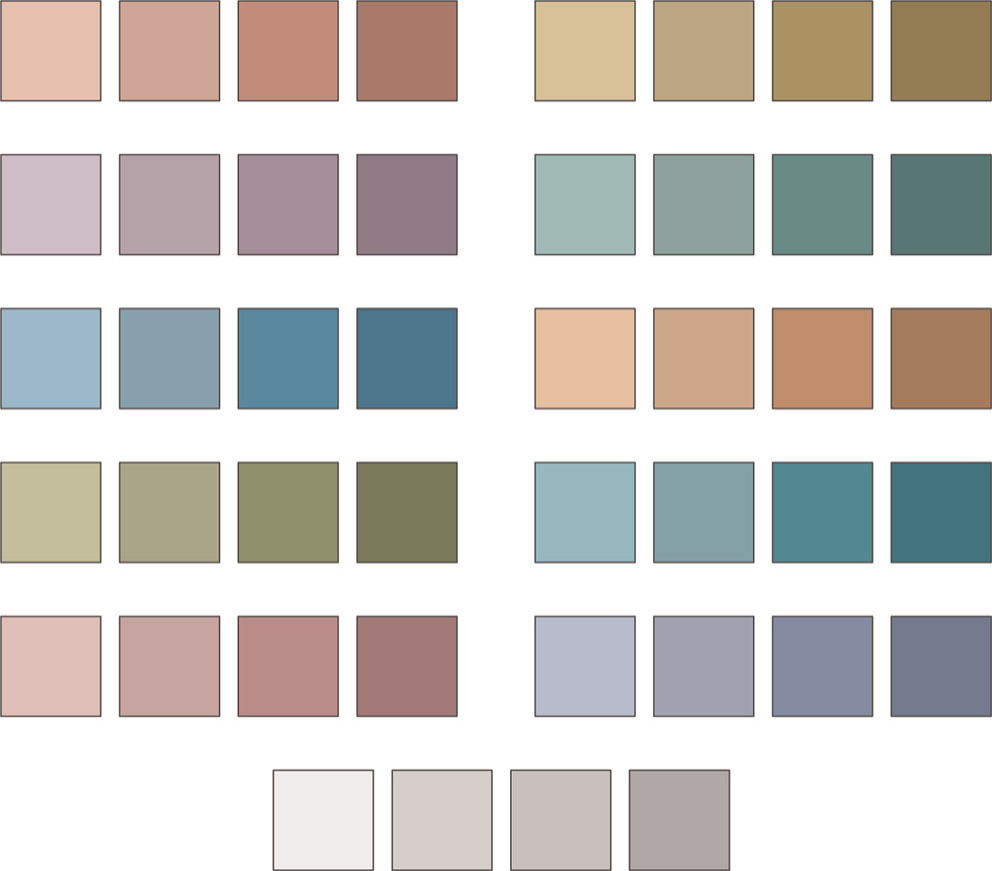
**Luminance color ramps for indicating protein conservation in the alignment**. 10 hues were chosen to be equally distinct from each other in a perceptual color space to represent the presence of a conserved domain on a protein; the same color is used for the same protein across domains. Each step along the color map (and the colormap for the background at the bottom) indicates an increase in the amino acid conservation on the protein at that position.

Unlike other systems for viewing alignments, MSAVis depicts an overview that iterates over conserved domains rather than the protein alignment; for example, there are five conserved domains or related structures in Figure 3 (Cyt_C5_DNA_methylase [CDD:cd00315], DNA_methylase [CDD:pfam00145], Dcm [CDD:COG0270], PRK10458 [CDD:PRK10458], and COG1092 [CDD:COG1092]). The horizonatal block for each domain repeats the alignment (Figure 4). The alignment position is displayed across the top. Initially, the entire sequence is shown; thus, individual amino acids may not be resolvable (potentially having been reduced to a few pixels or less of horizontal space). The strength of the alignment at any position is communicated with a luminance color ramp based on sum-of-pairs scoring at each position in the alignment provided by the user: At each position, BLOSUM62 [16] is used to score the alignment of each pair of amino acids; these are summed over all possible combinations at that position to calculate the conservation "strength" *s*. Given the maximum and minimum *s *over the entire alignment, we normalize the strengths to fall in the range [0, 1]. We use these to look up the corresponding color in a 256-entry colormap (Figure 5); the colormaps are designed such that a linear increase in the normalized *s *corresponds to a linear decrease in brightness (darker is more conserved). Thus, dark, saturated, contiguous regions are highly conserved while the lighter regions between them indicate mismatches or possible gaps. For example, in Figure 3, the conservation is not very strong (not a lot of dark grey), but not very poor either (not a lot of white). Currently, the range of luminance variance cannot be changed.

Each sequence in the alignment is assigned a color, as shown in the key at the top of the interface. Each protein color has roughly the same base luminance so that brightness changes due to the sequence strength appear consistent for each protein (Figure 5). In the visualization, a bar of color is drawn wherever the respective conserved domain is present in that sequence across a specified portion of the alignment. In Figure 3, it is easy to tell that the DNA_methylase site-specific domain is present in every sequence near both ends of the alignment. Similarly, the COG1092 domain (bottom) is only extant in human (green, [GenBank:NP_004403]) and chimp (purple, [GenBank:XP_001151907]). The depiction also quickly identifies potential incomplete domain classifications; for example, the Cyt_C5_DNA_Methalase domain (top group) is missing from the beginning of the Opossum protein whereas it is present in all eight other species. Furthermore, even though most of the domains overlap, it is still easy to see where they lie on the alignment since they are displayed on separate tracks.

The alignment overview can be useful for drawing general conclusions about some parts of the alignment. Often, however, this view will motivate the user to look more closely at an alignment subset. MSAVis allows the user to drag a rectangle around the area of interest to explore the conserved domains' relationship to the alignment in greater detail.

#### More detailed views

**Figure 6:**
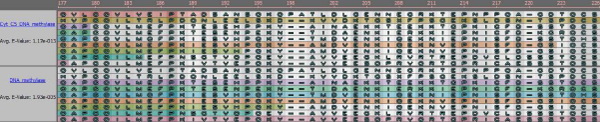
**MSAVis viewing a subset of the alignment**. Subsequences over the proteins can be zoomed in order to investigate potential causes for the alignment or positions of the conserved domains. At certain zoom level, the individual amino acids for a protein are displayed. In this example, we can see that the dissimilarity in alignment leads to a gap in DNA_methylase over several species. The expectation value displayed is provided by the CDD.

When zooming, the detail view smoothly replaces the overview (Figure 6). This view is very similar to the overview but is constrained to the sequence subset; the user can continue to select subregions as desired. When the user selects an area small enough to make drawing the individual amino acids characters viable, these are also displayed. Figure 6 shows a portion of the alignment where most of the sequences have the DNA_methylase domain present. In this view, a user can see each individual amino acid in every sequence in addition to the strength of the matches. In this example, it is easy to see that at position 187 (a quarter of the way from the left), all of the amino acids match in every sequence, so the colors are darkest. However, in the middle of the view, as the alignment strength lessens (gaps are inserted and columns are mismatched), the colors become lighter.

Users can scroll through the alignment using the scroll button on their mouse as well as the arrow keys on the keyboard. Additionally, users can click and drag the sequence labels on the left to move that sequence higher or lower in the stack, for closer analysis of a subset of the sequences, while still keeping information about all the sequences available. Similarly, domains may be removed entirely from the display by deselecting them from the checkbox of CDs near the top of the interface (Figure 3). Finally, users can easily zoom back out to the full view by clicking the right mouse button.

#### Visual and interaction design

Considerable care was taken in the visual design of MSAVis. For the proteins, unique, equal brightness colors were chosen; we choose ten hues roughly evenly separated in the CIE L*a*b* perceptually uniform color space [17]. In other words, each of the colors is equally visually distinguishable (barring color-blindness). The colors are the same brightness so that we can independently use luminance to encode the amount of conservation. Both of these visual cues are pre-attentive and separable [18] so that conservation strength and CD membership can be seen at a glance simultaneously.

The interaction with the interface also used cognitive principles in its design. Exploration of data is cyclic; thus, interfaces which facilitate overview-to-specific interactions mesh well with common exploration schemas [19]. Users can iteratively test hypotheses of the relationship between the alignment and the conserved domains – a user can zoom in on the gap in DNA_methylase first (Figure 6), and then examine the dissimilarity exhibited by COG1092 later. In addition, since our users will primarily be doing comparisons among proteins, the groupings of proteins into blocks facilitates this comparison. If a user wants to compare CDs, we do allow reordering of the CDs so two domains may be compared side-by-side. While this is not the most ideal arrangement, it is sufficient for pair-wise investigation.

#### Architecture

MSAVis is implemented in Python and uses BioPython [20] to load pre-aligned sequences in ClustalW or PHYLIP format and to calculate the sequence conservation at each location. This sequences are then processed live via the NCBI CD database to find conserved domains. It is cross-platform, using PyOpenGL [21] and wxPython [22] for its rendering and window management. We are currently considering a web-based option to interface with AgBase at Mississippi State [23]; the software is currently available from the AgBase website [24].

## Results and discussion

MSAVis provides a single view for presenting alignment conservation and the presence or absence of a conserved domain over a group of proteins. Unlike Jalview or the CDD, it depicts multiple conserved domains that may coincident over several proteins. This facilitates comparison of the conserved domains across species with less effort that previously available. MSAVis has both an overview and zoomed in view of the sequence (like Jalview), but contains these within a single display that can be dynamically navigated (unlike Jalview or the CDD). The overall goal of MSAVis is reduce the time required to explore the relationship between multiple proteins and their conserved domains and we have achieved this by providing both a compact view of this information and reducing the number of interactions with the data. For example, to compare the five conserved domains over the nine proteins given in Figure 3 would require nine views within the CDD (one for each protein) or 5 views in Jalview (one for each domain).

Feedback from our biologist colleagues has been positive, and we are deploying the tool for their use. Currently, the tool is for browsing only: It does not allow editing of the sequences or changing the alignments as does Jalview or other tools. Per the request of our collaborators, we are looking into this feature for the future.

Exploration using MSAVis is interactive; zooming in or out takes less than a second. Online access to the CDD and processing the domains when loading takes the most time (roughly a minute for the example presented in this paper).

Our tool provides a unique approach to the simultaneous display of the alignment and conserved domains that is not currently found in widely available tools such as MEME, GenDoc, and BioEdit. MEME [25,26] is primarily a tool for discovering sequence motifs but also provides a number of methods for displaying the discovered motifs (the information content at each position in the pattern, a logos format of the motif, and a neighbor-joining tree of the motif). The output most similar to that provided by our tool is a blocks diagram of the discovered motifs on the training set sequences; MEME does not support simultaneously viewing motifs and the alignment. GeneDoc [27,28] provides a set of tools for visualizing, editing, and analyzing multiple sequence alignments of both proteins and nucleic acids in an evolutionary context. These tools provides two residue display modes (display all or display only those different from the master sequence) that can be combined with a number of different gray-scale or color shading modes. There are also options allowing the user to define grouping and shading options that could be used with conserved domains imported from an external source such as NCBI. However, there is no easy mechanism for simultaneous visualization of domains and alignment strength nor for providing both detailed and high level views of the alignment. BioEdit [29] is primarily a sequence editing program but does also allow the user to import and display features from GenBank or GenPept sequences including the region feature that often corresponds to a conserved domain. However, all regions will be displayed at one time in one color on a single alignment. Our approach focuses on a small part of the larger protein sequence analysis problem that these popular tools address.

While MSAVis has proven successful for jointly visualizing sequence alignment and functional domain data, it has its limitations. As mentioned previously, we only have a limited set of unique hues for proteins; after ten, they begin to repeat. We made an attempt to stagger the colors so that similar hues are not next to each other, but this becomes more difficult as more than 12 proteins are added. In addition, as the number of conserved domains increase, the space used by the checkbox toggles increases; we are investigating more space efficient presentation for the CD toggles.

## Conclusion

Our prototype application demonstrates a new method for displaying multiple sequence alignments and conserved domains at different levels of detail. Both the alignment and the location of CDs can be viewed at a glance, and interactive exploration facilitates understanding of their interrelationship. We have taken a principled approach using results from visualization design to create an effective visualization. Initial feedback from our biology colleagues has been positive, and we are currently exploring their data for further system improvements.

As protein sequences become available for many non-model species, the need for mechanisms to analyze changes in conserved motifs will increase. For example, the availability of the opossum and platypus genomes provided insight into the functional motifs of mammalian genes. MSAVis provides a rapid method for investigating how these motifs are different from each other as more organisms are added.

In the future, we plan to investigate methods for displaying additional layers of information on the display, such as predicted DNA binding sites; some of this data is already present in CDD views but only for a protein at a time. In addition, we will explore a web-based version for distribution. More sophisticated methods scoring the alignment, such as information theoretic-based approaches, may also be explored to enhance the visual presentation. Finally, as mentioned, our collaborators have expressed the desire to be able to edit sequences; by editing the sequence and updating the conserved domain display, the accuracy of a sequence or its relationship to its domain might be better understood.

## Availability and requirements

• **Project name**: MSAVis

• **Project home page**: http://agbase.msstate.edu/tools/MSAVis.html

• **Operating system(s)**: Platform independent

• **Programming language**: Python

• **Other requirements**: Python 2.4–2.6, BioPython 1.5 or later, PyOpenGL 2.09 or later, wxPython 2.8 or later

• **License**: BSD

• **Any restrictions to use by non-academics**: Contact authors

## Competing interests

The authors declare that they have no competing interests.

## Authors' contributions

**TJK **Conceived and initiated the project. Designed the visualization, color coding, and interaction. Wrote the manuscript.

**ADL **Wrote and tested the software. Wrote an initial draft of the manuscript.

**SMB **Conceived and initiated the project. Provided feedback on the software design. Contributed to the manuscript.
